# Differences in Patterns of Alcohol Consumption Among Hispanics in the United States, by Survey Language Preference, Behavioral Risk Factor Surveillance System, 2005

**Published:** 2009-03-15

**Authors:** William S. Pearson, Shanta R. Dube, David E. Nelson, Raul Caetano

**Affiliations:** Behavioral Surveillance Branch, Division of Adult and Community Health, Centers for Disease Control and Prevention; Centers for Disease Control and Prevention, Atlanta, Georgia; Centers for Disease Control and Prevention, Atlanta, Georgia; University of Texas and University of Texas Southwestern Medical Center, Dallas, Texas

## Abstract

**Introduction:**

Alcohol consumption is pervasive in the United States, and extent of alcohol consumption for the growing US Hispanic population needs further study. We examined the association between language chosen for a national health survey and alcohol use among Hispanic adults.

**Methods:**

Hispanic participants aged 18 years and older (N = 20,234) from the 2005 Behavioral Risk Factor Surveillance System were stratified by choice of language (English, n = 13,035; Spanish, n = 7,199) for completing the survey. Differences for these 2 groups in current alcohol use, heavy alcohol use, and binge drinking were determined by using χ^2^ analyses and logistic regression models.

**Results:**

In bivariate associations, current drinking (*P* < .001), heavy drinking (*P* < .001), and binge drinking (*P* = .002) were significantly higher among participants who chose to complete the survey in English than among those who elected to complete the survey in Spanish. After controlling for demographic characteristics, associations between language choice and drinking behaviors were found to be greatest among women. Compared with women who chose to complete the survey in Spanish, women who chose to complete the survey in English were more than twice as likely to report current drinking (odds ratio [OR] = 2.42, 95% confidence interval [CI] = 2.02-2.91), heavy drinking (OR = 3.82, 95% CI = 1.44-10.10), and binge drinking (OR = 2.51, 95% CI = 1.64-3.84).

**Conclusion:**

This study suggests that language choice when completing a health survey is a predictor of high levels of alcohol use among Hispanic adults in the United States and that differences in drinking behaviors based on language choice for a survey are more profound among women.

## Introduction

Over the past 20 to 30 years, major demographic shifts in ethnic composition have occurred in the United States, particularly among the Hispanic population. From 1970 to 2000, the Hispanic population grew in the United States from 4.7% to 12.5% (1). Moreover, recent census data indicate that Hispanics are the fastest growing population in the United States, with an estimated total population of 42.7 million (2). Census data projections suggest that the number of Hispanics in the United States will continue to grow and will double by the year 2050 (3).

Many epidemiologic studies have been conducted to examine patterns of health and disease among this growing population, especially in an effort to reduce health disparities. Epidemiologic studies of alcohol use among Hispanics in the United States have indicated high estimates of general alcohol use and more hazardous alcohol-use patterns compared with other ethnic groups, although the levels were slightly lower than those for whites (4-12). Alcohol use was the third leading cause of preventable death in the United States in 2000 (13), and compared with other immigrant groups, Hispanics, especially men, have a greater propensity for hazardous alcohol consumption and suffer from more alcohol-related problems (14). For instance, white Hispanic men have the highest rate of cirrhosis mortality in the United States, compared with black and white non-Hispanic men, white Hispanic women, and black and white non-Hispanic women (15). Therefore, further research about the use of alcohol in the Hispanic population is relevant to public health.

The growth of the Hispanic population in the United States is creating a culture in which Spanish is spoken by 1 of 5 US citizens (2). Because of this shift, several studies have examined language preference among Hispanics as a predictor of health outcomes. For example, Spanish-language preference was found to be a barrier to the receipt of health care services (16) and a predictor for the receipt of less-efficient care (17). Conversely, comparisons of Spanish- and English-language preference have shown a higher prevalence of illicit drug use and smoking in groups that preferred English compared with Spanish (18,19). Furthermore, language of survey has been used as a 1-item measure of acculturation for smoking among Hispanics in a state-based population survey (20). Many other studies have examined the effects of acculturation on alcohol use and, in general, show that acculturation is related to higher rates of drinking, especially among women (5,21,22). Many studies show that acculturation has a general effect of liberalizing norms and attitudes toward drinking and, thus, increases drinking, but clear differences have been found between men and women (23-26). However, many of these studies had substantial limitations, which included low power due to small sample sizes (11) and data being collected in local areas (eg, single states, small number of treatment centers) and covariates not being sufficiently handled with the use of statistical tests (27).

The purpose of our study was to examine language preference for a health interview as a predictor of alcohol consumption patterns among Hispanic adults in the United States. We are not aware of any studies to date that have examined language preference solely as a predictor of alcohol consumption patterns in a nationally representative sample of Hispanics in the United States. Furthermore, we used a large, nationally representative sample and tests of statistical significance to overcome the limitations of previous studies and to confirm language preference as a predictor of alcohol use among Hispanic adults in the United States.

## Methods

Data for our study were taken from the 2005 Behavioral Risk Factor Surveillance System (BRFSS) survey. The BRFSS is an ongoing, state-based, random-digit–dialed, land-line telephone survey of adults aged 18 years or older that collects information on health risk behaviors, preventive health practices, and access to and use of health care services primarily related to chronic conditions. The data collected in the BRFSS are weighted to provide national estimates. In 2005, a total of 356,212 respondents participated in the survey. The median response rate for this survey was 51.1%, and the median cooperation rate, defined as the proportion of people interviewed of all eligible people who were actually contacted, was 75.1% (28).

The sample for this study was limited to participants who identified themselves as Hispanic and who resided in 1 of the 50 US states or the District of Columbia. The sample size for our analysis was 20,234, which represented approximately 28 million people in the United States. The sample was stratified by language preference of the survey respondents; group assignment was determined by whether the survey was conducted in Spanish (n = 7,199) or English (n = 13,035). Demographic characteristics examined for each group were age, sex, marital status, number of adults in the household, education level, employment status, and region of the country in which the participant lived.

Three measures of alcohol use were determined for this sample: current drinking, heavy drinking, and binge drinking. Current drinking was determined by the question, "During the past 30 days, have you had at least 1 drink of any alcoholic beverage, such as beer, wine, a malt beverage, or liquor?" People who responded no were not asked the subsequent questions on heavy drinking and binge drinking; thus, heavy drinking and binge drinking were assessed only among current drinkers. Heavy drinking was defined as having more than 2 drinks per day for men and more than 1 drink per day for women and was determined by the question, "One drink is equivalent to a 12-ounce beer, a 4-ounce glass of wine, or a drink with 1 shot of liquor. On the days when you drank, during the past 30 days, about how many drinks did you drink on the average?" (29). Binge drinking was defined as having 5 or more drinks on 1 occasion during the past 30 days and was determined by asking, "Considering all types of alcoholic beverages, how many times during the past 30 days did you have 5 or more drinks on 1 occasion?"(30). Respondents who chose "don't know/not sure" or "refused" as a response to any of the 3 questions were excluded from the analyses.

The sample was described by providing estimates of the different demographic characteristics (age, sex, marital status, number of adults in the household, education level, employment status, and region of residence) by language of the survey. Bivariate analyses were performed on the 3 alcohol measures between participants who chose to complete the survey in English and those who chose Spanish. Chi-square tests were used to analyze the independent association of alcohol consumption with language preference. Significance was set at α = .05. Separate logistic regression models were performed to examine the association between the 3 alcohol measures and language preference, while controlling for demographic variables. Both bivariate and multivariate analyses were conducted for the entire sample, as well as stratified by sex because of findings from previous research suggesting that the effects of acculturation on drinking patterns differ for men and women (31,32). All analyses were conducted using SUDAAN (RTI International, Research Triangle Park, North Carolina) software to account for the complex sampling design of the survey.

## Results

Participants who chose complete the survey in English and in Spanish were similar in terms of age and sex (Table 1). For both groups, approximately half of the population was 18 to 35 years of age, and both groups were nearly evenly divided between men and women.

Compared with participants who were interviewed in Spanish, a smaller proportion of participants interviewed in English reported that they were married or part of a couple (Table 1). Participants interviewed in English had higher levels of education than those interviewed in Spanish. In addition, participants interviewed in English had a much smaller proportion of households with more than 2 adults (36.4%) compared with participants interviewed in Spanish (53.0%). Participants interviewed in English were slightly more likely to indicate that they were employed compared with participants interviewed in Spanish (64.8% vs 61.2%). Finally, the geographic distribution of these 2 groups indicated that participants interviewed in English were much less likely than those interviewed in Spanish to be living in the West (39.2% vs 52.7%).

Statistically significant differences existed between the groups for the 3 alcohol consumption measures in bivariate relationships (Table 2). Among participants interviewed in English, 54.3% indicated that they had used alcohol within the past 30 days compared with 36.7% of those interviewed in Spanish (*P* < .001). Participants interviewed in English had a larger proportion of participants who were heavy drinkers (*P* < .001) and a larger proportion of participants who were binge drinkers (*P* = .002) compared with participants interviewed in Spanish.

Similar outcomes were found when focusing the analyses on men (n = 7,493) and women (n = 12,741) separately (Table 2). Among men, those who chose to be interviewed in English rather than Spanish had a significantly greater proportion of respondents indicating that they had used alcohol within the past 30 days (62.6% vs 53.6%, *P* < .001). Among women, those who chose to be interviewed in English rather than Spanish also had a significantly greater proportion of respondents indicating that they had used alcohol within the past 30 days (46.2% vs 18.3%, *P* < .001). For heavy and binge drinking, however, the differences between participants who chose to be interviewed in English and those who chose Spanish were significant only among the women. Among women, 4.2% of those interviewed in English reported heavy drinking compared with only 0.8% of those interviewed in Spanish (*P* < .001). Similarly, 8.6% of women interviewed in English reported binge drinking compared with 2.8% of women interviewed in Spanish (*P* < .001).

The logistic regression model for the combined sample of men and women demonstrated that, after controlling for demographic factors, participants who chose to complete the survey in English rather than Spanish were significantly more likely to have consumed alcohol within the past 30 days. Participants interviewed in English were nearly twice as likely to have engaged in heavy drinking and 40% more likely to participate in binge drinking compared with those interviewed in Spanish (Table 3).

Similar results were found when examining outcomes for men and women separately. Both men and women interviewed in English were significantly more likely than those interviewed in Spanish to report current, heavy, and binge drinking. Of participants who chose to be interviewed in English, odd ratios (ORs) among Hispanic women were larger than those for Hispanic men (Table 3).

## Discussion

The prevalence estimates and adjusted ORs for current, heavy, and binge drinking were higher among Hispanics who chose to complete the BRFSS in English than those who chose to complete the survey in Spanish. These results held true for both men and women separately, although the differences were particularly striking among women. To our knowledge, these are the most recent, nationally representative data stratified by language of survey on drinking patterns among Hispanics in the United States, and the results suggest that preferred language of survey administration is predictive of alcohol use in this population.

The finding that English-speaking Hispanic women were more likely to drink than were English-speaking Hispanic men confirms previous research on acculturation and substance abuse (17). Alcohol use has been a part of American culture for more than 300 years, and its use is a socially accepted behavior (33,34). As Hispanic women become more acculturated to American society, they are possibly more willing to participate in the social norms of the host society and less likely to feel the influence of traditional Hispanic culture (11,35). As has been previously noted, acculturated individuals tend to have more liberal attitudes (23). Therefore, they are more willing to participate in behaviors that previously had been taboo for them. Alternatively, Hispanic men in their native culture may have felt less societal pressure to abstain from drinking (36). These ideas could possibly explain why women who chose to complete the survey in English demonstrated a stronger proclivity to participate in drinking than did men.

Variations in drinking patterns among the heterogeneous Hispanic populations in the United States have been attributed to sex (37), country of origin (6), and level of acculturation (38,39). Acculturation has been a prominent focus of research on alcohol use among Hispanics (40,41). Acculturation involves changes in the beliefs, attitudes, and behaviors of immigrant populations as they adapt or assimilate to living in the dominant culture or society. Measurements of acculturation have typically included several questions such as country of origin, length of time spent in the host country, language preference, and feelings of interaction with the new culture (42,43).

Measuring acculturation in large population-based surveys such as the BRFSS can be challenging because the instruments used to measure the construct are lengthy. Large national surveys such as the BRFSS and the National Health Interview Survey, for example, collect data on many aspects of health and health care, which means there is competition for question space within the surveys. Given this challenge, the use of a 1-item question on language preference as a proxy measure may provide a feasible and accurate method to assess acculturation. A previous study of cigarette smoking behavior among US Latino men and women reported a high correlation (*r* = 0.8) between the 1-item question on language preference for the survey and a validated instrument used to measure acculturation (44).

Furthermore, researchers using the BRFSS in Oregon tested the 1-item question on language of survey to examine the effect of acculturation on smoking (20). Given that this measure has been used previously as a predictor of acculturation and was found to provide similar results to studies using more in-depth measures of acculturation, the use of this 1-item proxy in a national survey could save both time and money when conducting large numbers of interviews.

However, we are not advocating an indiscriminate use of proxy measures of acculturation in all research. Much research focusing specifically on the effects of acculturation and drinking should continue to employ longer and multi-item measures of acculturation. This will allow for a deeper understanding of how different dimensions of this construct (eg, language use, adoption of social norms, social interaction patterns, access to employment) are associated with drinking behaviors and how such associations change with different alcohol-related behaviors cross-sectionally and over time.

One practical implication of our study is the need to create materials in Spanish to reduce or prevent alcohol misuse. Alcohol industry representatives, noting the increasing number of Hispanics in the United States, have targeted this population (45). Alcohol product advertising and marketing campaigns in Spanish have been implemented, and a substantial proportion of Hispanic youth is being exposed to both English and Spanish alcohol advertisements (45). Therefore, from a public health perspective, Spanish language materials that discuss the effects of alcohol use should be used to target both Hispanic adults and youth. Providing interventions in Spanish may help the intervention more fully resonate with the Hispanic population and possibly provide primary prevention against alcohol misuse among Hispanics early in the acculturation process.

In our review of the literature, we found no studies that addressed preventing Hispanic women from adopting US alcohol use patterns during the acculturation process. Our study and others (42) suggest that interventions and further research in this area are needed.

Our study had some limitations that should be considered. First, the BRFSS is a land-line telephone survey, and not all people in the United States possess land-line telephones. A report from the US Census Bureau indicated that Hispanic households have a slightly lower rate of land-line telephone coverage than do white households (46). This situation could reduce the likelihood of Hispanics participating in this survey. Second, because findings are based on self-report, response bias may have been introduced into the results. Furthermore, underreporting of alcohol consumption is especially common among survey participants (47). Therefore, these estimates of drinking behaviors may be lower than the actual occurrence.

Despite these limitations, our analyses suggest that language of preference for survey interviews is associated with alcohol use among Hispanics in the United States. As previous work has suggested, language preference alone may be used as a reasonable proxy measure of acculturation. Future studies should consider language preference and its association with other health behaviors among Hispanics in the United States.

## Figures and Tables

**Table 1:** Demographic Characteristics of Hispanic Survey Participants (N = 20,234), by Survey Language Preference, Behavioral Risk Factor Surveillance System, 2005

Characteristic	Participants Interviewed in English, % (95% CI) (n = 13,035)	Participants Interviewed in Spanish, % (95% CI) (n = 7,199)	*P* Value^a^
**Age, y**			
18-35	49.7 (47.9-51.5)	52.4 (50.1-54.7)	.34
36-49	26.0 (24.6-27.4)	28.7 (26.8-30.7)	
50-64	16.0 (14.8-17.2)	11.7 (10.3-13.1)	
≥65	8.4 (7.4-9.4)	7.2 (6.0-8.4)	
**Sex**			
Male	49.4 (47.6-51.2)	52.0 (49.7-54.3)	.07
Female	50.6 (48.8-52.4)	48.0 (45.7-50.3)	
**Marital status**			
Married/part of couple	59.0 (57.2-60.8)	71.3 (69.2-73.4)	<.001
Ever married	15.3 (14.1-16.5)	12.7 (11.3-14.0)	
Never married	25.8 (24.0-27.6)	16.0 (14.1-18.0)	
**No. of adults in household**			
≤2	63.6 (61.7-65.6)	47.0 (44.9-49.1)	<.001
>2	36.4 (34.5-38.4)	53.0 (50.1-55.1)	
**Education level**			
Less than high school graduate	17.1 (15.7-18.5)	58.6 (56.5-60.7)	<.001
High school graduate	33.3 (31.5-35.1)	25.5 (23.6-27.5)	
Some college	28.6 (27.0-30.2)	10.4 (9.0-11.8)	
College graduate	21.0 (19.6-21.4)	5.5 (4.5-6.5)	
**Employment status**			
Employed	64.8 (63.0-66.6)	61.2 (59.1-63.3)	<.001
Unemployed	7.1 (6.1-8.1)	5.9 (6.9-7.9)	
Other	28.1 (26.5-29.7)	32.9 (30.8-35.0)	
**Region of country**			
Northeast	16.4 (15.2-17.6)		.05
South	34.5 (32.9-36.1)	28.5 (26.7-30.3)	
Midwest	9.9 (9.1-10.7)	5.7 (4.7-6.2)	
West	39.2 (37.4-41.)	52.7 (50.6-54.8)	

Abbreviation: CI, confidence interval.

a *P* values derived from χ^2^ test.

**Table 2:** Prevalence of Current^a^, Heavy^b^, and Binge^c^ Drinking Among Hispanics, by Survey Language Preference, Stratified by Sex, Behavioral Risk Factor Surveillance System, 2005

Drinking Status	Participants Interviewed in English, % (95% CI)(n = 13,035)	Participants Interviewed in Spanish, % (95% CI)(n = 7,199)	*P* Value^d^
**Overall**			
Current drinking	54.3 (52.5-56.1)	36.7 (34.4-39.0)	<.001
Heavy drinking	6.4 (5.2-7.5)	3.8 (3.0-4.6)	<.001
Binge drinking	18.0 (16.4-19.6)	14.4 (12.6-16.2)	.002
**Men (n = 7,493)**			
Current drinking	62.6 (59.9-65.3)	53.6 (50.1-57.1)	<.001
Heavy drinking	8.7 (6.8-10.7)	6.5 (4.7-8.3)	.11
Binge drinking	27.6 (24.9-30.3)	25.1 (21.8-28.4)	.25
**Women (n = 12,741)**			
Current drinking	46.2 (44.1-48.3)	18.3 (16.2-20.4)	<.001
Heavy drinking	4.2 (3.0-5.4)	0.8 (0.2-1.4)	<.001
Binge drinking	8.6 (7.2-10.0)	2.8 (2.0-3.6)	<.001

Abbreviation: CI, confidence interval.

a Current drinking was defined as having at least 1 drink of any alcoholic beverage during the past 30 days.

b Heavy drinking was defined as having more than 2 drinks per day for men and more than 1 drink per day for women during the past 30 days.

c Binge drinking was defined as having 5 or more drinks on 1 occasion during the past 30 days.

d *P* values derived from χ^2^ test.

**Table 3:** Adjusted Logistic Regression for Likelihood of Current, Heavy, and Binge Drinking Among Hispanics (N = 20,234)^a^, by Survey Language Preference, Stratified by Sex, Behavioral Risk Factor Surveillance System, 2005

Language Preference	Odds Ratio (95% CI)		

	Current Drinking^b^	Heavy Drinking^c^	Binge Drinking^d^
**Overall**			
English	1.75 (1.53-2.01)	1.96 (1.33-2.91)	1.41 (1.14-1.75)
Spanish	1 [Reference]	1 [Reference]	1 [Reference]
**Men (n = 7,493)**			
English	1.51 (1.22-1.86)	1.77 (1.11-2.82)	1.40 (1.08-1.79)
Spanish	1 [Reference]	1 [Reference]	1 [Reference]
**Women (n = 12,741)**			
English	2.42 (2.02-2.91)	3.82 (1.44-10.10)	2.51 (1.64-3.84)
Spanish	1 [Reference]	1 [Reference]	1 [Reference]

Abbreviation: CI, confidence interval.

a Adjusted for age, marital status, number of adults in the household, education, employment status, and region of the country.

b Current drinking was defined as having at least 1 drink of any alcoholic beverage during the past 30 days.

c Heavy drinking was defined as having more than 2 drinks per day for men and more than 1 drink per day for women during the past 30 days.

d Binge drinking was defined as having 5 or more drinks on 1 occasion during the past 30 days.
